# Influence of Covariates on ^18^F-FDG PET/CT Diagnostic Accuracy for Liver Metastasis

**DOI:** 10.3390/diagnostics14141466

**Published:** 2024-07-09

**Authors:** Vincent Habouzit, Anthime Flaus, Jean-Marc Phelip, Sylvain Grange, Bertrand Le Roy, Rémi Grange, Nathalie Prévot

**Affiliations:** 1 Department of Nuclear Medicine, University Hospital of Saint-Etienne, 42055 Saint Etienne, France; 2 Targeting Research Unit in Oncology, University Hospital of Saint-Etienne (URCAS), 42055 Saint Etienne, France; 3 Department of Nuclear Medicine, Hospices Civils de Lyon, 69500 Bron, France; 4 Medical Faculty of Lyon Est, University Claude Bernard Lyon 1, 69003 Lyon, France; 5 Department of Gastroenterology, University Hospital of Saint-Etienne, 42055 Saint Etienne, France; 6 Department of Radiology, University Hospital of Saint-Etienne, 42055 Saint Etienne, France; 7 Department of Surgery, University Hospital of Saint-Etienne, 42055 Saint Etienne, France; 8 SAINBIOSE U1059, INSERM, Mines Saint-Etienne, Université Jean Monnet Saint-Étienne, 42023 Saint Etienne, France

**Keywords:** fluorodeoxyglucose F18, positron emission tomography computed tomography, oncology, liver metastasis

## Abstract

(1) Background: ^18^F-FDG PET/CT diagnostic accuracy for liver metastasis (LM) could be influenced by technical parameters, lesion size, and the patient’s covariates. This retrospective study aimed to evaluate these covariates’ impact on PET/CT sensitivity. (2) Methods: Consecutive patients with suspected LMs who underwent ^18^F-FDG PET/CT scans were included. PET/CT scans were interpreted visually. The reference standard integrated histopathological and imaging follow-up. Logistic regression modeling and average marginal predictions were used to calculate per-lesion diagnostic performance measures with cluster robust 95% confidence intervals and to assess the covariates’ impact on PET/CT sensitivity. (3) Results: We included 192 patients with 330 lesions. ^18^F-FDG PET/CT exhibited a per-lesion sensitivity, specificity, positive predictive value, and negative predictive value of 86%, 79%, 91%, and 69%, respectively. In multivariate analysis, TOF PET/CT exhibited a higher sensitivity than non-TOF PET/CT (91% vs. 78%, *p* = 0.02). Sensitivity was reduced for lesions < 10 mm compared to larger lesions (56% vs. 93%, *p* < 0.001). A 5 kg/m^2^ increase in BMI led to an average 5% sensitivity reduction (*p* < 0.001). Age, sex, blood glucose level below 11 mmol/L, and liver density did not significantly impact sensitivity (*p* > 0.05). (4) Conclusions: ^18^F-FDG PET/CT sensitivity might be reduced with non-TOF PET, lesions < 10 mm, and higher BMI.

## 1. Introduction

The liver is the most common site of metastatic spread after lymph nodes. Colorectal, gastric, pancreatic, lung, and breast tumors and melanomas are among the cancers that most frequently metastasize to the liver [1]. Importantly, liver metastases (LMs) serve as a crucial prognostic factor that may significantly influence therapeutic decisions [2].

The primary imaging modalities for detecting LMs are contrast-enhanced Multiphase Computed Tomography (ceCT) and multimodal Magnetic Resonance Imaging (MRI) [3].

Diffusion-weighted MRI with gadoxetic acid-enhanced MRI provides the highest sensitivity for detecting LMs on a per-lesion basis [4]. Importantly, overall, MRI is more sensitive than CT in detecting LMs of less than 10 mm in diameter and can improve lesion detection in patients with underlying steatosis [5].

[^18^F]-fluorodeoxyglucose positron emission tomography (^18^F-FDG PET/CT) is a complementary imaging modality for LMs [6]. As a metabolic imaging technique, its diagnostic accuracy may be influenced not only by technical parameters and lesion size but also by patient-related factors [7]. While normal liver parenchyma generally exhibits mild and relatively consistent ^18^F-FDG uptake, factors such as patient age, blood glucose levels (BGL), body mass index (BMI), and underlying hepatic steatosis could influence FDG uptake in liver tissue [8,9,10,11,12]. The influence of these covariates on ^18^F-FDG PET/CT diagnostic accuracy for LMs remains understudied. This knowledge gap is particularly concerning given the expanding use of ^18^F-FDG PET/CT across oncological disciplines and the high incidence of hepatic metastases across cancer types. Acknowledging these limitations and understanding the intricate interplay between covariates could ultimately contribute to improving imaging protocols and diagnostic accuracy.

The main objective of this study was to evaluate the influence of lesion size, PET/CT camera, and patient-related covariates on the per-lesion sensitivity of PET/CT, using a modeling approach.

The secondary objective of the study was to assess the overall per-lesion diagnostic performance of ^18^F-FDG PET/CT for LMs.

## 2. Materials and Methods

### 2.1. Study Design and Patients

This single-institution retrospective study was approved by a French ethics committee for research on nuclear medicine (reference number: CEMEN 2023-03, date of approval: 28 November 2023). Informed consent was waived in compliance with national regulations pertaining to retrospective observational studies.

A report on adherence to The Standards for Reporting of Diagnostic Accuracy (STARD 2015) checklist is available in Supplement Material (Table S1).

A review of the database of our academic center was undertaken to identify consecutive cancer patients with suspected liver lesions from any imaging modalities (e.g., ultrasonography, CT, MRI, and PET/CT) who underwent ^18^F-FDG PET/CT between November 2015 and November 2019.

The inclusion criteria were as follows: (a) patients aged over 18 with non-liver primary neoplasms confirmed by histology and up to five suspected LMs; (b) ^18^F-FDG PET/CT performed for initial staging, systematic follow-up, or recurrence diagnosis, adhering to international PET tumor imaging guidelines (especially capillary glucose < 11 mmol/L) [13]; (c) no systemic therapy at least 4 weeks before PET/CT; (d) availability of abdominal ceCT or MRI performed within 6 weeks before or after the PET/CT scan, with no anticancer therapy during this interval; (e) availability of histopathology or morphological liver imaging follow-up of at least 6 months for LM diagnosis confirmation or rejection.

Only one PET/CT scan per patient was included: the first examination that met the inclusion criteria during the inclusion period.

### 2.2. PET/CT Protocol

Patients fasted for 6 h, maintaining glycemic control (capillary glucose < 11 mmol/L) before ^18^F-FDG injection. PET/CT acquisition occurred one-hour post-injection [13].

Two PET/CT cameras were used: the Biograph6^®^ LSO Pico 3D PET/CT HI-REZ (Siemens Medical, Erlangen, Germany), from November 2015 to May 2017, and the Biograph mCT Flow 20^®^ PET camera (Siemens Medical, Erlangen, Germany), from June 2017 to November 2019. These cameras are further referred to as Non-Time of Flight PET/CT (non-TOF PET/CT) and Time-of-Flight PET/CT (TOF PET/CT), respectively.

The protocol for the Biograph6^®^ LSO Pico 3D PET/CT comprised an intravenous injection of 4 MBq/kg of ^18^F-FDG, a low-dose non-enhanced CT acquisition (6 strips; 130 kV, 95 mAs; 5 mm slice thickness; pitch 1.45; caredose), and a PET acquisition (7 to 8 steps of 16.2 cm and 3 min each; OSEM reconstruction 4 iterations 8 subsets, 5 mm FWHM Gaussian post-filtering).

The protocol for Biograph mCT Flow 20^®^ included an intravenous injection of 3 MBq/kg of ^18^F-FDG, a low dose non-enhanced CT (20 strips; 120 mAs, 100 kV; 2 mm slice thickness; pitch 1.2; caredose), and a PET acquisition (TOF PET system, Flow 1 mm/s; OSEM reconstruction 2 iterations 21 subsets, 5 mm FWHM Gaussian post-filtering).

### 2.3. PET/CT Analysis

Two nuclear medicine physicians (V.H, A.F) with more than 5 years’ experience reviewed PET/CT scans in consensus, blinded by clinical data and other imaging results. Each visible focus of increased FDG uptake above the intensity of the liver background was recorded along with its location.

Confidence in the visual diagnosis was scored using a five-point scale: (1) definitively no metastasis, (2) probably no metastasis, (3) indeterminate lesion, (4) probably metastasis, and (5) definitively metastasis. Suspected LMs on ceCT or MRI performed within 6 weeks before or after the PET/CT scan but not recorded by the nuclear medicine physicians were subsequently scored as 1.

PET/CT was considered positive for lesions with scores of 4 or 5 and negative for lesions with scores of 1 or 2. For lesions with a score of 3, it was considered false positive or false negative regarding the reference standard (diagnostic misclassification for indeterminate PET/CT result).

### 2.4. Reference Standard

An abdominal subspecialized radiologist (R.G) and a nuclear medicine physician (V.H) reviewed morphological imaging (ceCT and MRI), along with clinical and biological follow-up data, to reach a consensus on the classification of lesions as either LMs or benign lesions (BL). The readers were unblinded from the PET/CT results to allow visual co-registration of lesion locations between imaging modalities. To avoid bias, metabolic change in lesions on PET/CT scans performed during follow-up was not considered as a criterion for lesion classification in the reference standard. Detailed criteria for the reference standard classification can be found in Appendix A.

### 2.5. Patients and Disease Characteristics

Data on gender, age, diabetes presence, capillary glucose level, BMI, primary cancer location, and previous treatments were recorded at the date of PET/CT examinations.

The number of suspected LMs and their size were recorded. The size of each suspected LM was determined by measuring the largest contrast-enhanced diameter on ceCT and/or MRI conducted within the 6 weeks after or before the PET/CT scans.

Liver density was measured on the non-enhanced CT coupled with PET by drawing a region of interest of at least 3 cm in diameter in an area of homogeneous liver parenchyma devoid of lesions. A mean liver density of 40 Hounsfield Units (HUs) or less was considered as hepatic steatosis [14].

### 2.6. Statistical Analysis

PET/CT sensitivity, specificity, positive predictive value (PPV), and negative predictive value (NPV) were calculated on a per-lesion basis.

Logistic regression modeling was used to determine measures of test accuracy. In summary, probabilities for the binary PET/CT result (set as the dependent variable) conditional on the reference standard (using separate datasets for LMs or BL) were computed, yielding values for sensitivity and specificity. Cluster-robust 95% confidence intervals (CI) were determined using the sandwich estimator of the variance to account for the clustering of multiple lesions per patient. The same approach was employed to calculate PPV and NPV as probabilities of the reference standard result conditional on the PET/CT binary result [15].

The PET/CT camera type, lesion size, and patients’ covariates were included as independent variables in the logistic regression model to evaluate their impact on PET/CT per-lesion sensitivity.

Initially, we conducted bivariate analyses, focusing on clinically relevant variables such as the PET/CT camera type (Non-TOF PET/CT or TOF PET/CT), lesion size (both as a continuous measure and categorized into three groups: no morphological lesion and lesions <10 mm or ≥10 mm), patient age (continuous), sex, BGL (continuous), BMI (both as a continuous measure and categorized as <30 kg/m^2^ or ≥30 kg/m^2^), and liver density (both as a continuous measure and categorized as ≤40 HU or >40 HU).

Then, a multivariate analysis was conducted, referred to as Model 1. This model included the PET/CT camera type and lesion size as categorical variables, while BMI, BGL, and liver density were treated as continuous variables. The selection of these variables was guided by their clinical relevance and the results of the bivariate analysis. These variables were included as continuous due to their observed linear relationship with the logit of the probability of the PET/CT result (Supplement Material Figure S1). Multicollinearity among explanatory variables was assessed using the Variance Inflation Factor (VIF). All covariates had VIF values under 1.5, below the acceptable threshold [16].

Finally, two alternative multivariate models were established. They were similar to Model 1 but they added interaction terms between the PET/CT camera type and lesion size (Model 2) and between the PET/CT camera type and BMI (Model 3).

Regression parameters (Beta coefficients), along with average marginal predictions and contrast differences in PET/CT positive results, were calculated in the restricted dataset of LMs for various covariate levels/categories [17]. These values represent sensitivities and sensitivity differences specific to each covariate level/category estimated by the model.

In the multivariate analysis, when assessing specific levels/categories of a variable of interest, unspecified covariate values were adjusted at their observed value [18].

The significance of the estimated sensitivity differences (null hypothesis: difference = 0) was tested, with cluster robust standard errors. The significance threshold was set at *p* < 0.05.

Analyses were performed using the statistical software R (R 4.3.0 GUI 1.79 Big Sur Intel build 8225 for Mac OS, The R Project) and the following packages: sandwich 3.0-2, marginaleffects 0.13.0, binom 1.1-1.1, car 3.1-2, lmtest 0.9-40, and ggplot2 3.4.2.

## 3. Results

### 3.1. Patients and Lesions Characteristics

**Figure 1:**
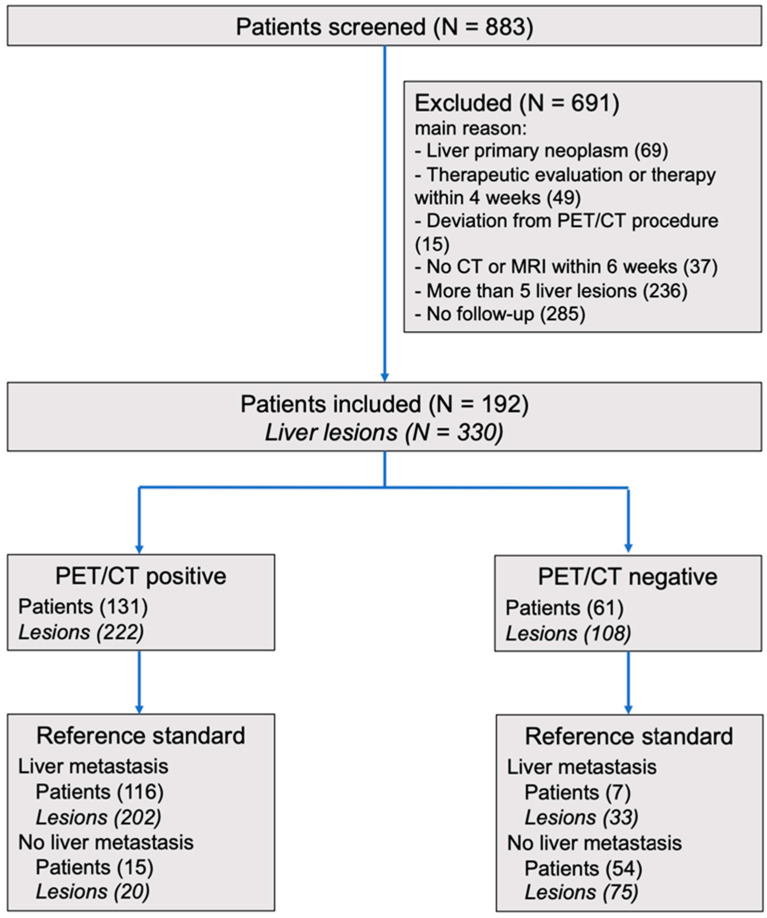
Study Flow Diagram; Abbreviations: CT, computed tomography; PET, positron emission tomography; MRI, magnetic resonance imaging.

We included 192 patients with 330 liver lesions (see Figure 1). An overview of the patients’ and lesions’ characteristics is shown in Table 1 and Table 2.

**Figure 2:**
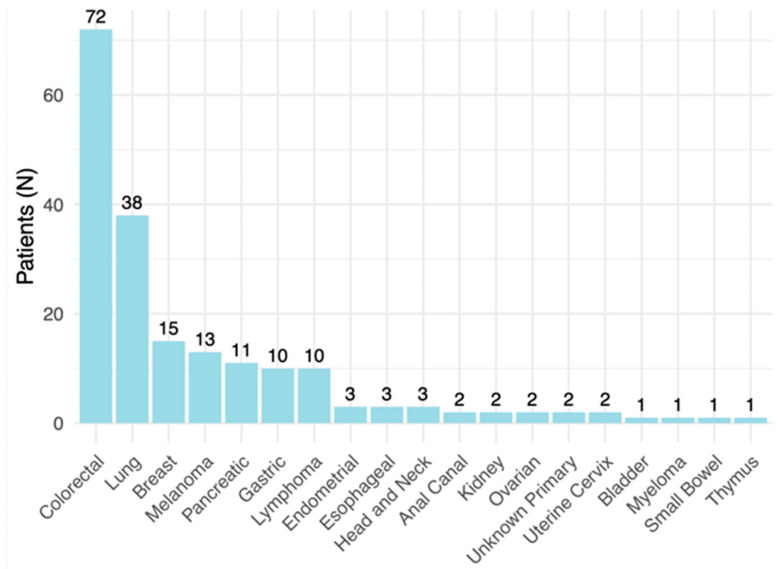
Primary neoplasms’ locations among patients.

The most prevalent primary cancer among the patients was colorectal cancer, accounting for 72/192 (37.5%) of the population (see Figure 2).

Histopathologic confirmation was available in 40/192 (20.8%) patients, covering 63/330 (19.0%) of the lesions. The median time interval between the PET/CT examination and histopathologic biopsy specimen or surgical resection was 45 days (range: 3–180 days). For other lesions, the classification relied on the morphological imaging follow-up combined with clinical and biological data. The median follow-up was 19.7 months (range: 6.1–56.5 months).

### 3.2. PET/CT Overall Diagnostic Accuracy

^18^F-FDG PET/CT exhibited a per-lesion sensitivity of 0.86 (95% CI: 0.80, 0.92), specificity of 0.79 (95% CI: 0.71–0.87), PPV of 0.91 (95% CI: 0.87, 0.95), and NPV of 0.69 (95% CI: 0.61, 0.78).

### 3.3. Variables Influencing the PET/CT Sensitivity

#### 3.3.1. Bivariate Analysis

##### Overall Results

**Figure 3:**
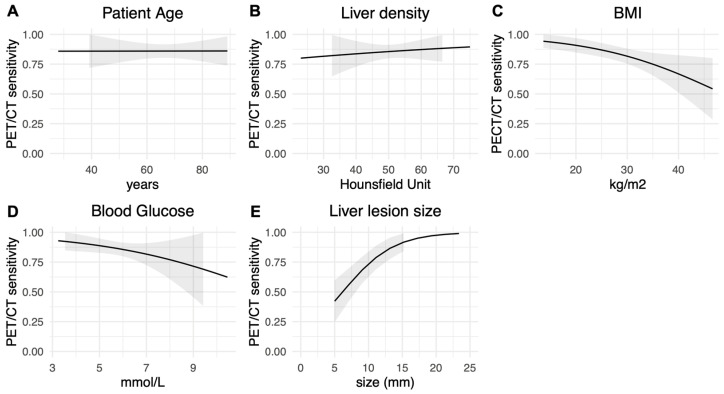
Graphical representation of the continuous covariates’ impact on the estimated overall PET/CT sensitivity in bivariate logistic regression analysis. Black line: Fitted average marginal predictions of PET/CT positive results in confirmed liver metastases, representing the estimated PET/CT sensitivity; Grey bands: cluster robust 95% confidence intervals. Abbreviations: CT, computed tomography; PET, positron emission tomography.

The results of the bivariate analysis are reported in Table 3 and Table 4. The impact of continuous covariates on PET/CT sensitivity is depicted in Figure 3.

TOF-PET/CT significantly enhanced the sensitivity compared to non-TOF PET/CT (*p* = 0.043).

In contrast, a reduction in lesion size and an increase in BMI were both associated with a decreased PET/CT sensitivity (*p* < 0.05). The impact of BMI on reducing PET/CT sensitivity seemed more pronounced for individuals with a BMI over 30 kg/m^2^, as illustrated by the steeper curve in Figure 3C. However, categorizing BMI as a two-class variable (BMI < 30 kg/m^2^ or ≥30 kg/m^2^) did not reach statistical significance (*p* = 0.069).

There was a trend toward decreased PET/CT sensitivity with lower liver density values (Figure 3B) and higher BGL (Figure 3D) but these effects remained statistically nonsignificant.

Finally, neither patient sex nor age had a significant impact on PET/CT sensitivity (*p* = 0.82 and *p* = 0.99, respectively).

##### Results in the TOF PET/CT Subgroup

In the TOF PET/CT subgroup, a reduction in lesion size and an increase in BMI were also associated with a decreased PET/CT sensitivity (*p* < 0.05). Patient sex, age, dichotomized BMI (BMI < 30 kg/m^2^ or ≥30 kg/m^2^), BGL, and liver density had no significant impact on the TOF PET/CT sensitivity (*p* > 0.05).

Detailed results of the bivariate analysis in the TOF PET/CT subgroup are reported in Supplement Material (Tables S2 and S3).

#### 3.3.2. Multivariate Analysis

Sex and patient age were not included in the multivariate analysis regarding the results of the bivariate analysis.

##### Overall Results

The results of the multivariate analysis of Model 1 are reported in Table 5.

The PET/CT sensitivity was significantly lower for lesions under 10 mm compared to larger lesions (*p* < 0.001). TOF-PET/CT increased the sensitivity (*p* = 0.020), whereas higher BMI was associated with decreased sensitivity (*p* < 0.001).

##### Results in the PET/CT Subgroups

**Figure 4:**
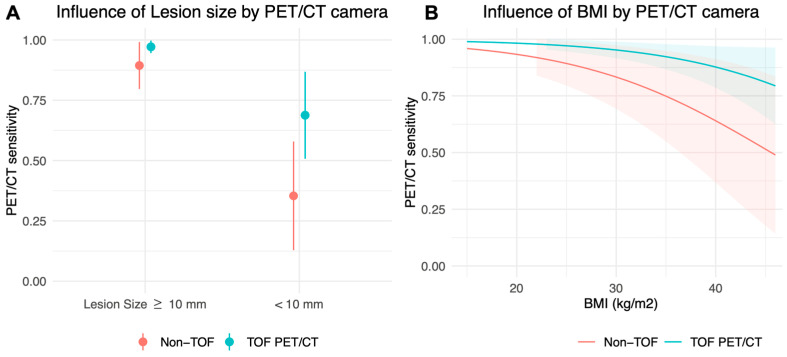
Influence of lesion size and BMI within PET/CT camera subgroups, from Model 1. (**A**) Dot: Average marginal predictions of PET/CT positive results in confirmed liver metastases, corresponding to estimated PET/CT sensitivity; Vertical bar: cluster robust 95% confidence intervals. (**B**) Color line: Fitted predictions of PET/CT positive results in confirmed liver metastases, corresponding to the estimated PET/CT sensitivity; Color bands: cluster robust 95% confidence intervals. Abbreviations: BMI, body mass index; CT, computed tomography; PET, positron emission tomography; TOF: Time of flight.

The impact of lesion size and BMI on PET/CT sensitivity remained statistically significant in both PET/CT camera subgroups. For lesions under 10 mm, sensitivity decreased by −0.29 (95% CI: −0.13, −0.45; *p* < 0.001) in the TOF PET subgroup and by −0.50 (95% CI: −0.321, −0.686; *p* < 0.001) in the non-TOF subgroup (Figure 4A), compared to larger lesions. An increase in BMI of +5 kg/m^2^ was associated with a PET/CT sensitivity decrease of −0.03 (95% CI: −0.05, −0.02; *p* < 0.001) in the TOF PET/CT subgroup and −0.06 (95% CI: −0.10, −0.03; *p* < 0.001) in the non-TOF subgroup (Figure 4B). Liver density and BGL did not have a statistically significant influence on the PET/CT sensitivity in both PET/CT camera subgroups (*p* > 0.05).

##### Multivariate Analysis with Interaction Terms between Covariates

Model 2 and Model 3 did not demonstrate any significant interaction between the PET/CT camera type and lesion size (Beta coefficient for the interaction term −0.178; 95% CI: −2.30, 1.94; *p* = 0.87) or between the PET/CT camera type and BMI (Beta coefficient 0.0278; 95% CI: −0.107, 0.163; *p* = 0.69), respectively. Model 1 exhibited greater parsimony compared to Models 2 and 3, as evidenced by the lower Akaike Information Criterion value of 153.18, compared to 157.01 and 155.01, respectively.

## 4. Discussion

In our study, ^18^F-FDG PET/CT scans were analyzed visually to align with routine clinical practice. Choi et al. conducted a meta-analysis on CRLM imaging diagnostic accuracy and reported a pooled PET/CT per-lesion sensitivity of 0.74 (95% CI: 0.62, 0.83) and specificity of 0.94 (95% CI: 0.84, 0.98) [4]. The authors identified heterogeneous accuracy among studies and used a meta-regression analysis to evaluate the impact of methodological covariates. They did not discuss the impact of lesion size, technical, and patient covariates on PET/CT diagnostic accuracy. Our study included a different population. Nevertheless, the slightly lower pooled sensitivity reported in the metanalysis could be attributed, at least in part, to the inclusion of mostly pre-2015 studies (9 out of 11) that may not have benefited from recent PET/CT technological advancements. Additionally, patients who underwent systemic therapy within the 4 weeks prior to PET were not included in our study, as this could lead to false negatives [19].

To the best of our knowledge, no other study had been conducted to evaluate the impact of covariates on PET/CT sensitivity for LMs using a multivariate modeling approach. In bivariate and multivariate analysis, we found three variables that might influence PET/CT sensitivity for LMs diagnosis: PET camera type, lesion size, and BMI.

TOF-PET/CT exhibited a higher sensitivity of 0.91 compared to 0.78 for non-TOF-PET/CT (*p* = 0.0204). This finding aligns with reported improvements in the image signal-to-noise ratio with TOF-PET [20]. Recent literature has also suggested improved lesion detection with digital PET/CT and total body PET/CT as well as image enhancement of non-TOF PET scans with deep-learning reconstruction methods [21,22,23].

Additionally, we found that lesion size below 10 mm had a substantial impact on reducing PET sensitivity, as reported in the literature [24]. This threshold was chosen because the detection and characterization of liver lesions smaller than 10 mm are considered clinically challenging in the literature [25]. Furthermore, the influence of lesion size remains significant when using a continuous variable in the bivariate model. This effect remained significant in both TOF and non-TOF PET/CT camera subgroups. The sensitivity for PET/CT detection of LMs below 10 mm was 0.56 in the multivariate analysis. This value is higher compared to that of 0.09 (95% CI: 0, 0.21) reported by Schulz et al. in a prospective study of CRLM before resection [26]. It could be attributed to the non-inclusion of very small metastases in our study, which could be only visible on pathological resection specimens or intraoperative ultrasound. Further research is needed to determine if combining digital PET/CT with deep-learning image reconstruction could improve the detection of small LMs [27].

We also found a decreased PET/CT sensitivity in higher BMI (*p* < 0.001). This is consistent with reported image degradation in PET among overweight patients. Protocol adjustments to enhance image quality in such patients have been proposed but they are usually not implemented in routine practice [28]. Furthermore, it has been shown that TOF-PET/CT could improve image quality for overweight patients [20,29]. However, in our study, a higher BMI was also associated with a decrease in TOF PET/CT sensitivity for LMs. Additionally, we did not observe a significant interaction between PET/CT camera type and BMI in Model 3; but, we cannot rule out the possibility of insufficient statistical power.

In both bivariate and multivariate analyses, neither BGL below 11 mmol/L nor hepatic density significantly impacted the PET/CT sensitivity. While a trend toward decreased sensitivity was observed with higher BGL and lower hepatic densities, the effect size was small. These findings suggest that these parameters may not play a substantial role in PET/CT diagnostic accuracy in routine clinical practice. This aligns with current PET procedure guidelines that recommend maintaining BGL below 11 mmol/L for optimal image quality [13], even though some previous studies have raised concerns about this range of BGL when the liver is the organ of interest [9]. Moreover, our results provide clinically relevant insights into the influence of hepatic steatosis on PET/CT diagnostic accuracy for LMs, addressing controversies in the literature regarding its impact on FDG liver uptake [8].

One strength of our study is the inclusion of a population representative of the routine clinical practice. This is, to date, one of the largest diagnostic accuracy studies of ^18^F-FDG PET/CT for LMs. Importantly, we specifically included patients with up to 5 suspected liver lesions, indicating an oligometastatic liver involvement. This focus on patients with a limited number of lesions enhances the clinical relevance of evaluating per-lesion diagnostic performance. In comparison, Schulz et al. included 342 lesions in 46 patients [26]. Identifying LMs at the oligometastatic stage is of utmost importance since their resection can be curative in the case of colorectal cancer [30,31]. Locoregional liver treatments for non-colorectal and non-neuroendocrine tumor metastases are not yet a standard of care. However, improved early detection of liver metastases could enhance patient selection that may benefit from these therapies [32].

Our study had a few limitations. Firstly, it is a retrospective study although we included a large population. As in most of the other retrospective diagnostic studies on LMs [4], histopathological evidence was only accessible for 19% (63/330) of the lesions. A more robust design for a prospective diagnostic study would be to conduct the index diagnostic test before hepatic surgery (with histopathological confirmation of resected lesions) coupled with intraoperative hepatic ultrasound and follow-up imaging of the remaining liver. This approach could enhance the classification of lesions and the detection of false negatives from the index test. Nevertheless, the use of a composite reference standard, with imaging follow-up of more than 6 months, remains relevant for classifying lesions as either LMs or BL. Furthermore, indeterminate PET/CT lesions were considered false negatives in order to provide conservative diagnostic performance measures, acknowledging the imperfect reference standard. Secondly, we included a heterogeneous population of primary cancer patients and we could not assess the influence of cancer phenotype factors in our model due to sparse data [33]. However, with a specific emphasis on evaluating the impact of lesion size and technical and patient covariates on PET/CT sensitivity for LMs, this population is still relevant. In this context, the influence of cancer phenotype may have been diluted and it should not have constituted a significant confounding factor.

## 5. Conclusions

^18^F-FDG PET/CT confirmed its high performance in detecting LMs. Moreover, we have shown that the sensitivity was notably reduced by non-TOF PET, lesion size below 10 mm, and high BMI. These findings could contribute to the refinement of imaging protocols and inspire the design of prospective trials aimed at improving PET/CT diagnostic accuracy, especially in challenging clinical scenarios.

## Figures and Tables

**Table 1:** Patients’ characteristics.

Characteristics	Patients (*N* = 192)
Age, years	68.0 (28.0–91.0)
Male	107 (55.7%)
Diabete	21 (10.9%)
Blood glucose level, mmol/L ^a^	5.5 (3.2–10.5)
BMI, kg/m^2 a^	24.5 (13.7–46.6)
BMI > 30 kg/m^2^	36 (18.8%)
Steatosis	24 (12.5%)
Number of liver lesion	
1	119 (62.0%)
2	32 (16.7%)
3	25 (13.0%)
4	8 (4.2%)
5	8 (4.2%)
Previous systemic therapy	71 (37.0%)
Previous local liver treatment	21 (10.9%)
TOF PET/CT camera	128 (66.7%)
PET/CT Clinical Indication	
Initial Staging	88 (45.8%)
Progression/Recurrence	91 (47.4%)
Systematic Follow-up	13 (6.8%)

^a^ Median (Range); *n* (%); Abbreviations: BMI, body mass index; TOF, time-of-flight; CT, computed tomography; PET, positron emission tomography.

**Table 2:** Lesions’ characteristics.

Characteristics	Benign Lesions (*N* = 95)	Liver Metastases (*N* = 235)
Lesion size, mm ^a^	12.0 (3.0–36.0)	16.0 (5.0–105.0)
Lesion size < 10 mm	12/48 (25.0%)	37/216 (17.1%)
No measurable lesion ^b^	47	19
Histological confirmation	3 (3.2%)	60 (25.5%)
MRI during follow-up	40 (42.1%)	120 (51.1%)
Lesion size during follow-up ^c^		
Increase	0 (0.0%)	137 (58.3%)
Decrease under treatment	0 (0.0%)	98 (41.7%)
Spontaneous decrease/disappearance ^d^	47 (49.5%)	0 (0.0%)
Stability	48 (50.5%)	0 (0.0%)

^a^ Median (Range); *n*/*N* (%); *n* (%); ^b^ No measurable lesion on contrast-enhanced abdominal CT or MRI performed within 6 weeks before or after the PET/CT examination, with no anticancer therapy during this interval; ^c^ Most informative criteria reported, see Appendix A for detailed criteria; ^d^ Decrease in contrast-enhanced lesion’s largest diameter or no morphological lesion visible on contrast-enhanced abdominal CT or MRI during follow-up, without any systemic therapy or local cancer therapy at that site; Abbreviations: MRI, magnetic resonance imaging; CT, computed tomography; PET, positron emission tomography.

**Table 3:** Bivariate logistic regression analysis: categorical variables’ influence on the estimated per-lesion overall PET/CT sensitivity.

Variable	Beta Coefficient	Sensitivity ^a^, Value (95% CI)	Sensitivity Difference ^b^, Value (95% CI)	*p*-Value ^c^
PET/CT camera				
Non-TOF	ref	0.78 (0.68, 0.88)	ref	
TOF	0.9837	0.91 (0.84, 0.96)	0.12 (0.01, 0.24)	0.043 *
Sex				
Female	ref	0.87 (0.78, 0.95)	ref	
Male	−0.1113	0.85 (0.78, 0.93)	−0.01 (−0.13, 0.10)	0.819
Liver density				
>40 UH	ref	0.87 (0.82, 0.93)	ref	
≤40 UH	−0.8831	0.73 (0.54, 0.94)	−0.13 (−0.34, 0.07)	0.199
BMI				
<30 km/m^2^	ref	0.88 (0.83, 0.94)	ref	
≥30 km/m^2^	−1.0795	0.72 (0.56, 0.89)	−0.16 (−0.34, 0.01)	0.069
Morphological Imaging				
Lesion ≥ 10 mm	ref	0.93 (0.88, 0.98)	ref	
Lesion < 10 mm	−2.4706	0.54 (0.37, 0.71)	−0.39 (−0.56, −0.22)	<0.001 *
No measurable lesion ^d^	−1.3113	0.79 (0.63, 0.95)	−0.14 (−0.31, 0.02)	0.088

^a^ Computed as average marginal predictions across covariate categories; ^b^ Computed as average marginal predictions contrast differences between covariate categories; ^c^ Testing estimated sensitivity difference = 0; ^d^ No measurable lesion on contrast-enhanced abdominal CT or MRI performed within 6 weeks before or after the PET/CT examination, with no anticancer therapy during this interval; Abbreviations: 95% CI, cluster robust 95% confidence intervals; PET, positron emission tomography; CT, computed tomography; ref, reference category; TOF, time of flight; BMI, body mass index; HU, Hounsfield Units; *, *p* < 0.05.

**Table 4:** Bivariate logistic regression analysis: continuous variables’ influence on the estimated per-lesion overall PET/CT sensitivity.

Variable	Beta Coefficient	Sensitivity Difference ^a^, Value (95% CI)	*p*-Value ^b^
Patient Age, +10 years	0.0002	0.01 (−0.05, 0.05)	0.99
Lesion Size, +1 mm	0.2668	0.02 (0.01, 0.03)	<0.001 *
BMI, +5 kg/m^2^	−0.07936	−0.05 (−0.07, −0.02)	0.001 *
Blood Glucose level, +1 mmol/L	−0.2869	−0.03 (−0.08, 0.01)	0.155
Liver Density, +5 HU	0.0144	0.01 (−0.03, 0.05)	0.639

^a^ Computed as the average marginal predictions contrast differences between covariate categories; ^b^ Testing estimated sensitivity difference = 0; Abbreviations: 95% CI, cluster robust 95% confidence intervals; PET, positron emission tomography; CT, computed tomography; ref, reference category; TOF, time of flight; BMI, body mass index; HU, Hounsfield Units; *, *p* < 0.05.

**Table 5:** Multivariate analysis from Model 1: variables’ influence on the estimated per-lesion overall PET/CT sensitivity.

Variable	Beta Coefficient	Sensitivity ^a^, Value (95% CI)	Sensitivity Difference ^b^, Value (95% CI)	*p*-Value ^c^
Categorical				
PET/CT camera				
Non-TOF	ref	0.78 (0.68, 0.88)	ref	
TOF	1.39254	0.91 (0.87, 0.95)	0.13 (0.02, 0.24)	0.02 *
Morphological Imaging				
Lesion ≥ 10 mm	ref	0.93 (0.88, 0.98)	ref	
Lesion < 10 mm	−2.73613	0.56 (0.41, 0.71)	−0.37 (−0.53, −0.21)	<0.001 *
No measurable lesion	−1.27456	0.81 (0.68, 0.94)	−0.12 (−0.26, 0.02)	0.087
Continuous				
BMI, +5 kg/m^2^	−0.10313	_	−0.05 (−0.07, −0.02)	<0.001 *
Blood Glucose, +1 mmol/L	−0.33174	_	−0.03 (−0.07, 0.01)	0.175
Liver Density, +5 HU	−0.03641	_	−0.02 (−0.04, 0.01)	0.117

^a^ Computed as the average marginal prediction across covariate categories; ^b^ Computed as the average marginal prediction contrast differences between covariate categories/levels; ^c^ Testing the estimated sensitivity difference = 0. Abbreviations: 95% CI, cluster robust 95% confidence intervals; PET, positron emission tomography; CT, computed tomography; ref, reference category; TOF, time of flight; BMI, body mass index; HU, Hounsfield Units; *, *p* < 0.05.

## Data Availability

The datasets used and/or analyzed during the current study are available from the corresponding author upon reasonable request. The data are not publicly available due to ethical restrictions.

## Supplementary Materials

The following supporting information can be downloaded at: https://www.mdpi.com/article/10.3390/diagnostics14141466/s1. Table S1: Adherence to The Standards for Reporting of Diagnostic Accuracy (STARD 2015) checklist; Table S2: Bivariate logistic regression analysis, categorical variables influence on estimated per-lesion TOF PET/CT sensitivity; Table S3: Bivariate logistic regression analysis, continuous variables’ influence on estimated per-lesion TOF PET/CT sensitivity; Figure S1: Linear relationship between continuous covariates and the logit of the probability of PET/CT positive result.

## Appendix A. Detailed Criteria Used for the Reference Standard Classification

Criteria for classifying a lesion as a liver metastasis (LM) were the following, in descending priority:

(a) Histopathologic results from liver resection or biopsy;
(b) Increase in contrast-enhanced lesion’s largest diameter by more than 20% (with a minimum of 5 mm change in size) or decrease by more than 30% for patients undergoing systemic therapy (with a minimum of 5 mm change in size);
(c) Typical LM appearance on both contrast-enhanced CT and MRI;
(d) Lesion stability under treatment (not meeting the above criteria) with typical signs of LMs on at least contrast-enhanced CT or MRI;
(e) Concomitant assessment of the overall disease course on imaging at other sites and on clinical and biological reports.

Criteria for classifying a lesion as a benign lesion (BL) were the following, in descending priority:

(a) Histopathologic results from hepatic resection or biopsy;
(b) Decrease in contrast-enhanced lesion’s largest diameter by more than 30% (with a minimum of 5 mm change in size) or no morphological lesion visible on contrast-enhanced CT and MRI follow-up without any systemic therapy or local cancer therapy at that site;
(c) Typical BL appearance on both contrast-enhanced CT and MRI;
(d) Lesion stability (contrast-enhanced lesion’s largest diameter decreased by less than 30% or increased by less than 20%) in the absence of cancer treatment or in contrast to the disease course under treatment on imaging at other sites and/or on clinical and biological reports.
