# Supplementary material for: Influence of Covariates on 18F-FDG PET/CT Diagnostic Accuracy for Liver Metastasis
Source: Diagnostics (Basel). 2024 Jul 9;14(14):1466. doi: 10.3390/diagnostics14141466 (PMC11276173; doi:10.3390/diagnostics14141466)

SUPPLEMENTARY MATERIAL

Table S1. Adherence to the Standards for Reporting of Diagnostic Accuracy (STARD 2015) checklist

Section & Topic	No	Item	Reported on page #
**TITLE OR ABSTRACT**			
	**1**	Identification as a study of diagnostic accuracy using at least one measure of accuracy (such as sensitivity, specificity, predictive values, or AUC)	Yes #1
**ABSTRACT**			
	**2**	Structured summary of study design, methods, results, and conclusions (for specific guidance, see STARD for Abstracts)	Yes #2
**INTRODUCTION**			
	**3**	Scientific and clinical background, including the intended use and clinical role of the index test	Yes #3, 4
	**4**	Study objectives and hypotheses	Yes #4
**METHODS**			
Study design	**5**	Whether data collection was planned before the index test and reference standard were performed (prospective study) or after (retrospective study)	Yes #4
Participants	**6**	Eligibility criteria	Yes #4
	**7**	On what basis potentially eligible participants were identified (such as symptoms, results from previous tests, inclusion in registry)	Yes #4
	**8**	Where and when potentially eligible participants were identified (setting, location and dates)	Yes #4
	**9**	Whether participants formed a consecutive, random or convenience series	Yes #4
Test methods	**10a**	Index test, in sufficient detail to allow replication	Yes #5, 6
	**10b**	Reference standard, in sufficient detail to allow replication	Yes #6
	**11**	Rationale for choosing the reference standard (if alternatives exist)	No
	**12a**	Definition of and rationale for test positivity cut-offs or result categories of the index test, distinguishing pre-specified from exploratory	Yes #5, 6, 11
	**12b**	Definition of and rationale for test positivity cut-offs or result categories of the reference standard, distinguishing pre-specified from exploratory	Yes # 6, 13
	**13a**	Whether clinical information and reference standard results were available to the performers/readers of the index test	Yes #5
	**13b**	Whether clinical information and index test results were available to the assessors of the reference standard	Yes #6
Analysis	**14**	Methods for estimating or comparing measures of diagnostic accuracy	Yes #7, 8
	**15**	How indeterminate index test or reference standard results were handled	Yes #6
	**16**	How missing data on the index test and reference standard were handled	NA
	**17**	Any analyses of variability in diagnostic accuracy, distinguishing pre-specified from exploratory	NA
	**18**	Intended sample size and how it was determined	No
**RESULTS**			
Participants	**19**	Flow of participants, using a diagram	Yes #Fig. 1

	**20**	Baseline demographic and clinical characteristics of participants	Yes #Table 1
	**21a**	Distribution of severity of disease in those with the target condition	Yes #Table 2
	**21b**	Distribution of alternative diagnoses in those without the target condition	No
	**22**	Time interval and any clinical interventions between index test and reference standard	Yes #9
Test results	**23**	Cross tabulation of the index test results (or their distribution) by the results of the reference standard	Yes #Fig. 1
	**24**	Estimates of diagnostic accuracy and their precision (such as 95% confidence intervals)	Yes #9
	**25**	Any adverse events from performing the index test or the reference standard	No
**DISCUSSION**			
	**26**	Study limitations, including sources of potential bias, statistical uncertainty, and generalisability	Yes #11, 12, 13
	**27**	Implications for practice, including the intended use and clinical role of the index test	Yes #11, 12, 13
**OTHER INFORMATION**			
	**28**	Registration number and name of registry	Yes #4
	**29**	Where the full study protocol can be accessed	No
	**30**	Sources of funding and other support; role of funders	Yes #19

Table S2. Bivariate logistic regression analysis, categorical variables influence on estimated per-lesion *TOF* PET/CT sensitivity

Variable	Beta coefficient	Sensitivity[a], Value (95% CI)	Sensitivity difference[b], Value (95% CI)	p-value[c]
Sex				
Female	ref	0.88 (0.78, 0.96)	ref	
Male	0.558	0.93 (0.87, 0.98)	+0.05 (-0.05, 0.10)	0.332
Liver density				
> 40 UH	ref	0.92 (0.87, 0.97)	ref	
≤ 40 UH	-1.22	0.77 (0.54, 0.99)	-0.15 (-0.38, 0.08)	0.212
BMI				
< 30 km/m2	ref	0.93 (0.88, 0.97)	ref	
≥ 30 km/m2	-1.28	0.78 (0.61, 0.95)	-0.15 (-0.32, 0.03)	0.103
Morphological Imaging				
Lesion ≥ 10 mm	ref	0.97 (0.93, 0.99)	ref	
Lesion < 10 mm	-2.75	0.64 (0.34, 0.84)	-0.33 (-0.53, -0.12)	0.001*
No measurable lesion[d]	-1.70	0.83 (0.62, 0.99)	-0.14 (-0.35, 0.08)	0.228

[a] Computed as average marginal predictions across covariate categories; [b] Computed as average marginal predictions contrast differences between covariate categories; [c] Testing estimated sensitivity difference = 0; [d] No measurable lesion on contrast-enhanced abdominal CT or MRI performed within 6 weeks before or after the PET/CT examination, with no anticancer therapy during this interval; Abbreviations: 95% CI, cluster robust 95% confidence intervals; PET, positron emission tomography; CT, computed tomography; ref, reference category; TOF, time of flight; BMI, body mass index; HU, Hounsfield Units; *, p < 0.05

Table S3. Bivariate logistic regression analysis, continuous variables influence on estimated per-lesion *TOF* PET/CT sensitivity.

Variable	Beta coefficient	Sensitivity Difference[a], Value (95% CI)	p-value[b]
Patient Age, +10 years	0.0209	0.02 (-0.01, 0.04)	0.17
Lesion Size, +1 mm	0.457	0.03 (0.02, 0.04)	<0.001*
BMI, +5 kg/m2	-0.0822	-0.04 (-0.07, -0.02)	0.035*
Blood Glucose level, +1 mmol/L	-0.857	-0.06 (-0.10, 0.03)	0.167
Liver Density, +5 HU	-0.0302	-0.01 (-0.05, 0.03)	0.588

[a]Computed as the average marginal predictions contrast differences between covariate categories; [b]Testing estimated sensitivity difference = 0; Abbreviations: 95% CI, cluster robust 95% confidence intervals; PET, positron emission tomography; CT, computed tomography; ref, reference category; TOF, time of flight; BMI, body mass index; HU, Hounsfield Units; *, $p < 0.05$

Figure S1. Linear relationship between continuous covariates and the logit of the probability of PET/CT positive result

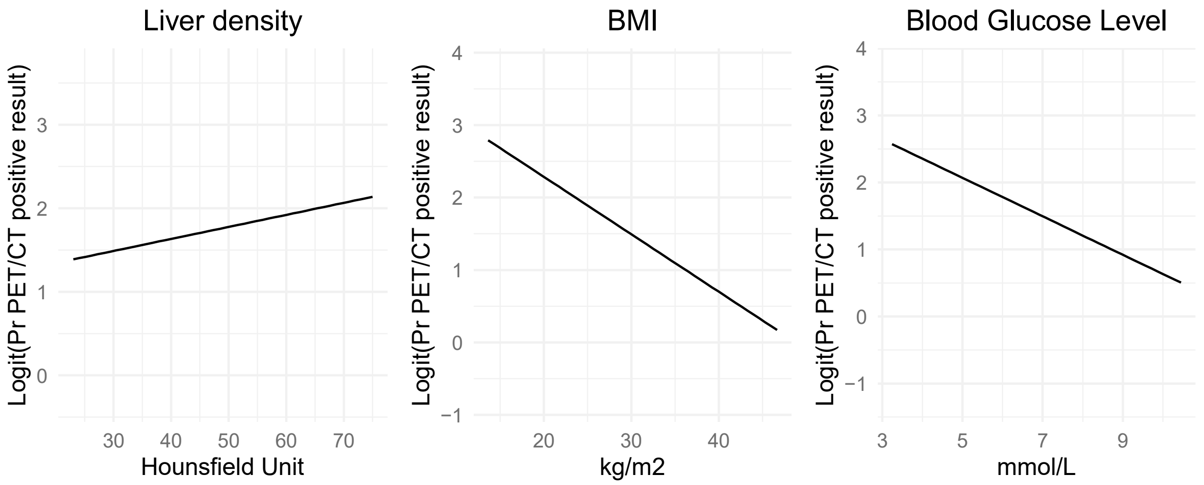

Supplement: Supplementary file 1 [file diagnostics-14-01466-s001.zip › diagnostics-3062133-supplementary.pdf]
